# Healthy community-living older adults’ experiences with using a specially adapted virtual reality exercise game to promote physical activity—a pilot study

**DOI:** 10.3389/fspor.2025.1665943

**Published:** 2025-12-10

**Authors:** Bård Bogen, Martin Moum Hellevik, Gro Gujord Tangen, Lars Peder Bovim, Nina Øye, Kristin Taraldsen

**Affiliations:** 1 Department of Global Public Health and Primary Care, University of Bergen, Bergen, Norway; 2 Stiftelsen CatoSenteret, Son, Norway; 3 The Norwegian National Centre for Ageing and Health, Vestfold Hospital Trust, Tønsberg, Norway; 4 Department of Geriatric Medicine, Oslo University Hospital, Oslo, Norway; 5 SimArena, Western Norway University of Applied Sciences, Bergen, Norway; 6 Department of Rehabilitation Science and Health Technology, Faculty of Health Sciences, Oslo Metropolitan University (OsloMet), Oslo, Norway

**Keywords:** virtual reality, exercise, game, older adults, qualitative

## Abstract

**Background:**

Immersive technologies such as virtual reality (VR) that has been developed the recent years, are now increasingly accessible and offers new opportunities for VR environments combined with games that may enhance physical activity. However, these games may require adaptation and further customization for use among older users. In this pilot study, we investigate the experiences of older adults using a head-mounted VR exercise game that was developed in close collaboration with end users.

**Methods:**

We included ten community-dwelling older adults aged 65 years and older who undertook six VR exercise sessions across a two-week period, each session lasting 15 min. Descriptive information included age, gender, mobility (Timed Up and Go test, TUG), and gait speed (10 m walk test), and daily steps from ankle-worn accelerometers. Semi-structured individual interviews were conducted, transcribed verbatim and analyzed by use of reflexive thematic analysis.

**Results:**

We included four men and six women, age range from 66 to 77 years, with an average gait speed of 1.4 m/sec and an average TUG-time of 7.6 s. Their average activity levels were close to 4200 steps per day. After the trial period, participants’ experiences were that VR is a new and exciting development. They found VR to be more of a game than exercise, and they suggested integrating more challenging activities, physical movement and social engagement to enhance the exercise part of the game. They did not feel that the game facilitated any more physical activity in their daily lives.

**Discussion/conclusion:**

VR is a promising tool that older adults in this trial enjoyed, but the game was not physically demanding enough for the participants. Future adaptations should include more challenges. Game development should focus on tasks that keep players interested and engaged over longer times, without jeopardizing safety.

## 1 Introduction

As longevity increases, the population composition in most European countries is increasingly made up of older adults (1). While this is a success of health care and healthier lifestyles, the burden on health care services is already apparent, with even higher burdens to be expected in the decades to come^1^. Consequently, measures that can contribute to sustained health, independence and quality of life in older age are needed. One of the most important single factors for healthy aging is physical activity and exercise, which can reduce the risk of all-cause mortality (2), frailty (3), the risk of several cardiovascular and metabolic diseases (4), and incident dementia (5), increase bone strength (6), and reduce falls (7). However, many older adults do not exercise and are not sufficiently physically active (8), and engaging in and keeping up an exercise routine can be difficult. Reasons for not exercising can include lack of interest, not believing that exercise could prolong your life, and feeling breathless and out of shape (9), while characteristics of exercise programs that promote adherence include individualization of exercise, education about the exercise program, the scientific rigor of the exercise selection, convenience, enjoyment, feedback and integration into daily life (10). Hence, the need for exercise programs that are fun, engaging and motivating, and that have lasting effects on physical activity.

Over the last decades, video games have been used increasingly for exercise purposes, in such cases termed as ‘exergaming’, which can be broadly defined as video games that “involves physical exercise and that integrates motion-tracking technology that enables interaction with the game and real-time feedback of user's performance” (11, 12). Benefits of exergames are that they can be tailored to the user's specific needs, with demands placed on different cognitive tasks or motor tasks according to relevant impairments (13, 14), but also that the gaming aspect is enjoyable, motivating and engaging (15). Importantly, the combination of cognitive and motor training (‘motor-cognitive training’) is hypothesized to facilitate additional neuroplastic effects for both domains (16). Studies over the last years suggest that exergaming can be more effective than conventional exercises for improving balance (17), cognitive functioning (18) and reducing falls (19) in older adults. A recent study showed that it should be noted there are also studies that show little or no difference to conventional exercises (14, 20). Exergames can be delivered over several different platforms, such as screens, surround-screen displays (21) or head-mounted displays (HMD). By using head-mounted virtual reality (VR) technology, all visual stimuli except what is shown in the display is removed. For this reason, this experience is generally considered the most immersive, and by extension, most engaging. There are few studies that compare outcomes of exergaming with HMDs to other platforms, but memory performance could potentially be better with HMDs (22).

Through a multidisciplinary collaboration funded by the EU-Active and Assisted Living program (23), a prototype of a novel VR-exercise game especially adapted for older adults has been developed. The goal of the collaboration is to develop an affordable VR solution which older adults can use by themselves at home to improve or maintain their levels of physical activity. Novel technological applications need to be specially adapted to the needs and abilities of the designated end users (24). However, as Song et al. (2025) points out: “*Current VR Game designs often follow generic models, lacking personalized adjustment to address individual differences in physical abilities and cognitive levels”.* Furthermore, research into VR and older adults often fail to sufficiently incorporate feedback from older adults during the design phase, resulting in systems that do not fully meet their needs (25, 26). Therefore, researchers are pointing to the need to gain better understanding about how older adults themselves experience using novel technology, such as VR, as tools to gain or support healthy ageing (24, 27, 28).

Therefore, the primary aim of this study is to investigate how older adults experience using a novel and technologically advanced VR-exercise game, and further whether playing the game would lead to more physical activity.

## 2 Materials and methods

### 2.1 Design

This was a pilot usability study where participants completed a two-week period of six sessions with a VR game developed for older adults. Data were collected in 2023 and 2024. The project was approved by Sikt (08.03.2023, Ref.nr. 458242). All participants signed a written informed consent prior to inclusion in the study.

### 2.2 Setting

The VR game sessions took place at a rehabilitation centre (CatoSenteret). Although the aim is that the game could be played at home, we chose to undertake the initial tests in a controlled setting for safety purposes.

### 2.3 VR game—SenopiPhy

As part of a larger European study (CoSoPhyFX (project number AAL-2020-7-215-CP), an immersive VR-game called SenopiMed was developed through several steps. SenopiMed is aimed at motor-cognitive exercise for older adults (29, 30). The development of SenopiMed was an interdisciplinary process which included researchers and clinicians (psychologists, psychiatrists, physiotherapists and sports therapists) in collaboration with older adults from Finland (*n* = 15) and Switzerland (*n* = 30). Using SenopiMed's software solution as a foundation, a separate version aimed specifically at physical exercise (called SenopiPhy) was developed. This version was adapted by the second author in collaboration with software developers from SenopiMed and then tested on three older adults in Norway. Based on the feedback from these sessions, a second version of the SenopiPhy was created for this pilot study.

**Figure 1:**
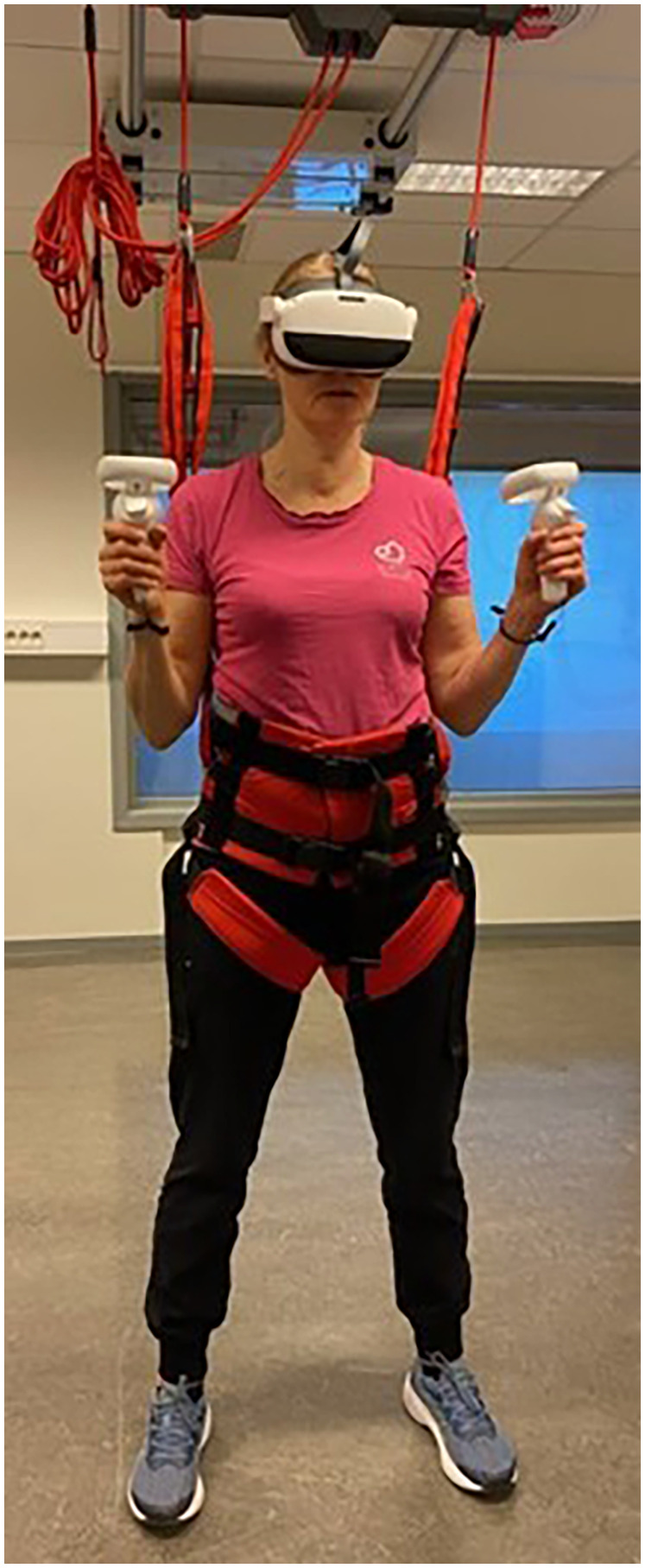
Person not included in the study showing the setup of the game (the person consented to use of image).

**Figure 2:**
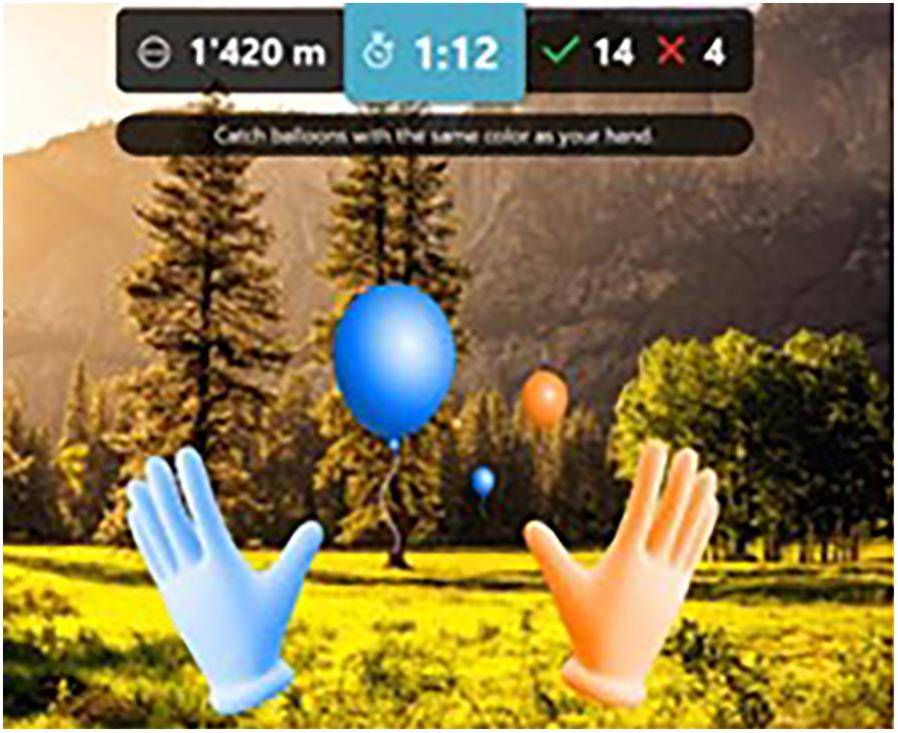
Screengrab of the game.

The goal of the SenopiPhy is to hit orange and blue balloons moving towards the player using motion controllers held in each hand (see Figures 1, 2). The motion controllers are visually seen as either orange or blue hands in the game, and in order to be hit the balloons successfully, the blue balloons have to be hit with the blue hand, and opposite. The objects appear at different areas in the visual field in front of the player and move at different speeds towards the player. The player's performance is rated by the number of objects the player can hit with the motion controllers within each session. If the player correctly hits ten objects successfully in a row, they will receive a visual and audio feature in the form of applause. The game is designed to challenge balance, coordination and cardiovascular capacity, while the player is in a standing position in 360 degrees immersive surroundings. The game has three levels of difficulty, with changing speeds, distances and angles of the ballons. The angle, distance and speed are preset for each difficulty. When the player is starting a session, he/she can choose from a variety of visual surroundings (beach, mountain, urban and forest) and different songs (classical, jazz, rock and pop). The HMD device used for this study was the Pico Neo 3 Pro.

### 2.4 Participants

Target population for this study was healthy older adults aged 65 years or older who are physically inactive. The inclusion criteria are presented in Table 1.

**Table 1:** Criteria for inclusion.

Inclusion criteria	Exclusion criteria
Community-dwelling older adults (65 years or older).	Conditions or disorders that contraindicates use of Head Mounted Displays (HMD), such as epilepsy, skin disorders that worsens when using the HMD, visual disorders or suffering from VR-induced motion sickness when using the VR-system.
Self-reported independence in daily life activities (ADL).	
Self-reported physically inactive, defined as not achieving or exceeding 150 min of moderate intensity physical activity weekly.	
Able to stand independently for 30 min.	
Able to wear a safety harness during the exercise sessions.	
Willing to use accelerometer (StepWatch4™) for objective measurement of stepping activity for 4 consecutive weeks.	
Speak fluent Norwegian.	
Able to follow verbally given instructions.	
Available for participation for a period of four consecutive weeks (including post- and pre-intervention measurements in addition to the intervention period).	

### 2.5 Recruitment

Potential eligible participants were invited from a municipality in the south-east region of Norway, through advertisement in the municipality's senior counselling service, the care service, social media, local senior centres, and word of mouth. We screened those contacting us via telephone and invited those fulfilling the inclusion criteria to participate.

### 2.6 Procedure

After inclusion, participants wore ancle-worn accelerometers for one week prior to baseline assessments prior to starting the first VR game session at the multidisciplinary rehabilitation centre. The pilot period lasted for two weeks in total with six 15 min VR game sessions. Before each session, participants were given 15 min to prepare for the initiation of the exercise, while the clinician helped with the equipment and starting the software correctly. After each session, participants were given 15 min to relax or change clothes. The final VR game session was followed by a face-to-face individual interview. The ankle-worn accelerometers were worn for the entire period and removed approximately one week after the final VR game session. It should be noted that the aim of the study is to investigate the usability of the game and that two weeks is unlikely to produce any real changes in physical activity. We still chose to include some measures of physical activity and physical function to be able to detect any changes.

### 2.7 VR game sessions

The VR game was tested with three versions, where the participants completed three game sessions in the same and increasingly difficulty order. The participants selected their preferred music (jazz, pop, electronic or rock) and type of visual scenery (mountain, beach, forest, or city/urban) for each VR game session. All participants wore the HMD while in a standing position with their shoes on and with a safety-harness securely attached to their trunk during the VR game sessions. A clinician supervised the sessions.

### 2.8 Outcome measures

#### 2.8.1 Background information

We collected descriptive information about the participants’ age, sex, marital status (yes/no), living arrangement (live with someone/live alone), employment status, and educational level. Measures of physical function included performance-based tests from the physical performance screening section of the European Working Group on Sarcopenia in Older People (EWGSOP) consensus algorithm (31). We used the Timed Up and Go Test (32) as a measure of mobility (seconds) and the five times sit to stand test (5xSTS) (33) as a functional measure of physical function (seconds). We measured grip strength with Jamar digital dynamometers, using the average performance from three tests on both right and left side (reported in kg). We calculated gait speed from the 10 m walk test (10MWT) (34) conducted at preferred speed using the average gait speed from two trials (m/sec). TUG, 5xSTS and 10MWT was measured at the same day as session one, before session one.

#### 2.8.2 Intensity and performance of the VR game sessions

Data regarding exercise intensity during each session was collected both with sensors and self-reporting. Objective measures of exercise intensity were collected using a Polar OH1 optical heart rate sensor attached to the upper arm, deriving data on maximum heart rate and average heart rate during each session. Self-reporting of exercise intensity was collected using the Borg Rating of Perceived Exertion Scale (35), with reporting right before and immediately after each VR game session.

Data regarding performance in the VR-exercise game was collected after completion of each session and included the number of successful hits of balloons during each session.

#### 2.8.3 Daily steps before, during and after the pilot period

We used ancle-worn accelerometers to measure daily steps by use of the StepWatch™ 4 accelerometer (SW4; Modus Health, Inc., Washington, DC) continuously for the entire trial period (four consecutive weeks in total). Walking is one of the most common forms of physical activity for older adults (36), and the StepWatch4™ is a valid and reliable accelerometer for measurement of stepping activity in older adults (37, 38). Data on number of mean daily steps was collected from the week before, the two-weeks during, and the week after the VR game sessions.

The SW4 is a small (75 × 48 × 14 mm) lightweighted (41 g) ankle-worn accelerometer, attached with a strap above the lateral malleolus.

#### 2.8.4 Interview data

We used individual, semi-structured interviews designed to obtain detailed responses from participants, providing valuable insights into their experiences and perceptions of the VR game. After developing the first version of the interview guide, it was critically assessed by the research team and one clinician, as well tested by two older adults representing the target population for this study. The combined feedback from these processes led to the development of the final interview guide (see Table 2).

**Table 2:** Interview guide questions.

Semi-structured interview, main questions	
1	To start with, can you tell me what motivated you to participate in this research project?
2	Now that you have completed six VR training sessions, can you share your experience of combining VR and exercise?
3	If you had the opportunity to continue using this VR training game on your own at home, would you do it?
4	Imagine you were one of the developers tasked with improving this game. What would you change?
5	In this research project, we aimed to recruit healthy individuals aged 65 or older who are physically inactive in their daily lives. Can you tell me a bit about what you enjoy doing in your daily life?
6	During the two weeks you participated in this study, you had three VR training sessions per week. How did you feel this affected your physical activity level in your daily life?
7	Lack of motivation is a significant challenge when it comes to becoming more physically active, and this seems to apply to most age groups, including those aged 65 and older. Do you have any thoughts on whether this VR training could increase motivation to be more physically active?
8	If you were to create a VR training game that you believe could motivate you to be more physically active, what would you do similarly or differently compared to this project's VR game?
9	Finally, do you have any other comments, thoughts, or experiences related to this project that you would like to share?

### 2.9 Data analysis

The data was analysed without grounding in any specific paradigm or perspective. The aim of the study was to gather user experiences regarding a specific item (the game), and we deemed the topic to be suited to a content analysis.

We processed all quantitative data using MS Excel and presented data by use of descriptive statistics. All coding was done manually, and no artificial intelligence software was used in transcription or analysis. The qualitative interview data were analysed using Braun and Clarke's six-phase approach for reflexive thematic analysis (39), involving repeatedly going back and forth from the raw data, via coding and clustering. The first (BB) and last (KT) authors conducted the analysis. Both, particularly KT, have previous experience with interview data, both from published studies and from supervision of master students. First, the first and last author read the raw interview data and noted initial ideas to familiarize with the data. The first impression was discussed between the two authors, and then again in a meeting with the second author who was the person conducting all interviews. Secondly, the last author generated initial codes by grouping the entire dataset into meaningful groups. Thirdly, the last author searched for themes by collating codes into potential themes. Fourthly, the themes were reviewed by going back and forth from the codes, initial themes, and potential themes. Fifthly, the themes were defined, and the essence of each theme was discussed among the first and last author. A few initial themes were excluded in this step. Finaly, the report was written and revised among the authors. An example of the coding process during the analysis is presented in Table 3.

**Table 3:** Example of the coding process during the analysis.

Quotes	Code label	Code group	Initial themes
*"No, I thought it sounded a bit exciting. It was just pure curiosity, really, yes."*	Exiting and I was curious	Fun to experience Virtual Reality	Exploring new concepts
*"Variation on multiple levels, not just variation in movements, but also in choice options. As much variation as possible. And also in intensity."*	There should have been greater variation across several levels	What could have been improved	Proposed changes

The number of respondents was decided before initiation of the study, and level of saturation in the interviews was not considered. It is therefore possible that further interviews would bring new information, but our clear impression was that the respondents shared fairly similar experiences.

## 3 Results

### 3.1 Participant characteristics

We included six women (66–76 years) and four men (67–77 years) with a mean age of 71.2 years (SD 4.2). All were married and lived with someone. All but one were retired from work, whereas the youngest woman still worked in a full position. Eight individuals held a higher education degree, while two possessed a vocational certificate. The males (*n* = 4) had on average a grip strength of 41.3 kg (SD 5.9) vs. 23.9 (SD 4.5) for the females (*n* = 6). Both men and women had almost identical average values for TUG (women 7.65 s, SD 1.61, men 7.63 s, SD 1.64) and gait speed (women 1.38 m/s, SD 0.22, men 1.38 m/s, SD 0.29). Functional strength, as shown by seconds on 5STS, was 9.6 (SD 2.2) vs. 12.0 (SD 2.9) for men and women. Individual participants’ characteristics are presented in Table 4.

**Table 4:** Participants’ individual characteristics.

Participants	M77	M67	M69	M70	F66	F76	F70	F74	F76	F67
Grip strength, kg	48.2	34.5	43.6	39.0	23.9	15.2	26.6	26.1	24.1	27.5
TUG, sec	10.0	6.1	6.1	8.3	8.4	10.8	5.9	7.4	6.5	6.9
5STS, sec	9.2	8.4	12.9	8.1	12.4	17.1	12.8	10.1	9.2	10.4
Gait speed, m/sec	1.3	1.8	1.0	1.3	1.3	1.0	1.7	1.3	1.5	1.5

M, male; F, female, and numbers indicating years of the participants.

TUG, time (sec) on the timed up and go test; 5STS, time (sec) on the 5 reps sit-to-stand test; Gait speed, gait speed (m/sec) from the 10 m walk test at preferred instructed speed.

### 3.2 Testing of the virtual reality game (intensity and performance of the VR game sessions)

During the two-week pilot period, seven participants completed all six and three participants completed five out of six VR exercise sessions. Overall, 57 sessions were completed with a total of 24 725 balloon hits. The average maximum heart rate across the sessions was 102 BPM. Overall, the participants rated their subjective perception of physical exertion at the beginning and the end of the sessions as 6.7 and 10.1 on the Borg Rating of Perceived Exertion (RPE) scale, with an average increase of 3.4 during each session (see Table 5).

**Table 5:** Performance and intensity of the VR game sessions. .

VR game session	Lowest level^a^		Medium level^b^		Top level^c^	
	Mean	SD	Mean	SD	Mean	SD
HITS	414.8/419	3.6	463.6/472	16.1	423.3/428	11.5
Max BPM	101.4	9.7	102.0	6.8	102.6	11.2
Average BPM	86.9	7.4	88.2	7.9	90.8	10.0
Borg pre	6.7	1.2	6.7	1.0	6.7	0.8
Borg post	10.2	1.8	10.1	1.7	9.9	1.7
Borg change	3.6	1.7	3.4	1.8	3.2	1.7

a VR game session 1 and 4.

b VR game session 2 and 5.

c VR game session 3 and 6.

**Table 6:** Objectively measured steps per day.

Daily steps	Before exercise period		During exercise period		After exercise period	
	Mean	SD	Mean	SD	Mean	SD
Steps	4,189.1	(1,727.4)	4,884.6	(1,830.0)	4,433.7	(2,169.3)
Range	2,519.3–7,620.7		3,017.1–8,375.0		4,764.7–7,721.3	

Daily steps = Number of steps per day from objectively ankle-worn accelerometers.

**Table 7:** Initial themes, sub-themes and main themes. .

Initial themes	Sub-themes	Main themes
Exploring new concepts	Exploring virtual reality was a pleasing experience Games Involve competition	Virtual reality represents a new and exciting development
Enjoyable involvement		
Competitive drive		
Game rather than exercise	Excessively simplified and oriented towards play Virtual reality provides minimal effect	Virtual reality is more oriented towards gaming than training
Expected more		
Does not affect overall physical activity levels		
Experienced effects		
Proposed changes	Needs to incorporate additional challenges and physical movements Social interaction would have provided motivation	Virtual reality should integrate more challenging activities, physical movement, and social engagement
Social engagement enhances motivation		

### 3.3 Daily steps before, during, and after the exercise period

Participants wore ancle worn accelerometers continuously during the study period, with a total of 44 registration days before (*n* = 7), 80 registration days during (*n* = 9), and 52 registration days after (*n* = 8) the two-weeks of VR exercise sessions. Table 6 presents the objectively collected daily steps for the participants before, during, and after the two-weeks exercise period.

### 3.4 Experiences (interview data)

All 10 participants completed the interview session at the end of the two-week pilot period. We developed three main themes from the interviews. Table 7 presents an overview of main themes and sub-themes.

#### 3.4.1 Virtual reality represents a new and exciting development

All participants described that they were excited and wanted to take part in this new and exciting development. One explained that he was a bit unsure what he was going to be part of, and wondered “*How will this unfold?”* (P4).

##### 3.4.1.1 Exploring virtual reality was a pleasing experience

Most participants highlight that this has been a pleasing experience, and that they were happy to join exploring the VR game. They used positive words such as “absolutely great” (P3), “an experience” (P8) “exciting” (P1) “pleasant” (P2), “enjoyable» (P4), «positive experiences» (P6), «all good» (P10), «fun» (P5). A very few explained that they had relatable experience with programming or playing games, but that this VR game was new to all of them. One person explained that she found it fun to be part of something that was in development, and one other said that in addition to being fun it was also felt good to contribute to this project.

“I think that being in these different environments is very rewarding. It's an experience, and with the music, I find it fun. So, I would say that if I were sitting in a nursing home and hadn't experienced much, it would be the highlight of my day.” (P8)

“Enjoyable. Yes, I woke up the next morning after the first time and thought, what am I doing today? And then I knew I was going to [activity], and I felt the desire to do it. So that fear of what I had gotten myself into disappeared after the first time…."(P4)

##### 3.4.1.2 Games involve competition

Several of the participants talked about games triggering competition and a feeling of wanting to achieve. One person described that she did not think of herself as a competitive person but realized that she probably was after testing the VR game. Some suggest that the developers should have included more measurements within the VR game along with feedback, as they find this motivating. Others suggest that the VR game could have included motivating games where more than one person could have participated.

“Yes, because everyone likes to be measured by something. You go to school, and there are grades. If there weren't, you wouldn't bother going to school. It's strange, really, we are driven by. It's the same with exercise, ‘Why do you do exercise?’ Well, to be the first to finish.” (P9)

#### 3.4.2 Virtual reality is more oriented towards gaming than training

The participants were highly positive towards the VR game, but at the same time emphasized that this VR game was too simple, more oriented towards gaming and not exercise, and provided minimal physical effects. As one person explained “*It becomes more of a game*” (P5).

##### 3.4.2.1 Excessively simplified and oriented towards play

All but one of the participants found the VR game excessively simplified, and several explained that it was more oriented towards play not exercise. Most said that they found the VR game very simple, and one even said that it was at the simplest level. Nevertheless, they liked the game, as one explained:

“Childish, yes, but it was incredibly fun to be childish, right? Yes, exactly. Play, it awakens something. They say you stop playing because you grow old. But you grow old because you stop playing. And I think that is very important.“ (P6)

The participants described different pre-expectations to the VR game. One underlined that he (P3) thought that it would be more challenging than experienced, but that he at the same time found the VR game to challenge his concentration. Others thought it would be more movement of both upper and lower extremities.

“I don't really think it's that much exercise, actually. I'm just standing there still.” (P5)

“I saw them more as a game and didn't really.. if I'm going to exercise, I want to get sweaty and warm. And I didn't get that here. I got a little sweaty on my forehead, but that was from the rubber ring on the headset. But I do see that there is potential for developing this into a form of exercise, absolutely. And if you could maybe involve the legs and so on, then yes, definitely.” (P1)

“Yes, because you can really see this as a game with red and blue balloons. So, if you're sitting in a chair and exercising at the same time, there are surely some people who would want to do that."(P2)

In line with this, most of the participants did not see themselves as the target population for this VR game and suggested that for example day center users would have been more appropriate.

##### 3.4.2.2 The game provides minimal physical effects

Overall, most participants experienced minimal physical effects of the VR game, including both immediately effects and transferred effects to daily physical activity levels. As one person explained: “*No, it [daily physical activity level] was not affected. I haven't done any more or less because of it."(P10)*

One person said that participation in the study led her to walk to the rehabilitation center for the game sessions. As such, it was not the game itself that led to increased physical activity, but the transportation back and forth, still she frames the activity in a positive way and puts it in the context of the game:

“Physically active? Well, maybe it made me want to be. Because when I get up in the morning, I often sit and watch the news, drink coffee, and knit, so after a while, I feel the need to move. So for me, it was a good opportunity to take an extra walk when I came here on those days. I probably wouldn't have done it otherwise. And then I got a walk on my way here and often on my way back home as well. And I thought that was alright, but beyond that, I didn't do many other exercises at home than what I naturally do throughout the day.” (P7)

#### 3.4.3 Virtual reality should integrate more challenging activities, physical movement, and social engagement

After six sessions using the VR game, the participants had several suggestions for improvements as well as suggesting add-on's to the VR game.

##### 3.4.3.1 Needs to incorporate additional challenges and physical movements

Most participants suggested to increase speed and variation of the movements that were part of the VR game, along with adding movements for the lower extremities. They really wanted more exercise and thus more challenges. A few suggested different rackets and stick sports, such as hockey, tennis and badminton. Some of the participants describe more progression, both in terms of level of difficulties and also how the visualization could be altered such as smaller balloons), and some suggested longer sessions when necessary due to progress. Overall, they wanted more movements, as they experienced the VR game to be a bit static as one described “*I feel glued to the floor"(P7)*.

“….I would have wanted it to be a bit more challenging. I would have liked to use my legs as well. That way, you would improve coordination and also get a higher heart rate.” (P8)

“More balloons. More switching up and down. Down up, from deep down to suddenly up. Then it becomes, well, then it becomes, yes, then it becomes squats [stands up from the chair and demonstrates]. Yes, then it becomes exercise.” (P1)

Additionally, one important input from our participants was that some felt discomfort with some of the virtual surroundings within the VR game. Several participants described a feeling of not being safe at the top of the mountains, and a sense of discomfort. As one explained:

“I got the feeling of being pulled outward, and I felt my hands getting sweaty. Yes."(P2)

##### 3.4.3.2 Social interaction would have provided motivation

Four of the participants underlined that regular exercise often involve something social, that this VR game is missing at the moment. As one person described, motivation from someone else could have been useful during exercising, and that they probably would not have played this game at home, on their own. They also emphasized that games cannot replace human interaction.

“Exercise is also a bit social for me. I need a bit of social interaction.” (P1)

“And then there's the issue of motivation to engage in physical exercise. Yes, I'm a bit unsure about how motivated I would be to do this at home daily. I don't think so; it really depends on the software.” (P1)

“…then I might have found it fun, especially if it was connected with something social, like you mentioned something at a senior gathering. So, if you met once a week, you would have this in common, some interaction or something, so you would have something to share with others when you met?” (P2)

## 4 Discussion

In this pilot study, we aimed to learn about the experiences of inactive older adults with an immersive VR exergame. Ten older adults were interviewed after the trial period and shared that they found the game interesting and visually pleasing, and that the overall experience had been fun. However, several also emphasized that the game was not very physically challenging, and that they saw it more as a game than as exercise.

The participants found the game to be novel, fun and interesting. Motivation is crucial for adherence to exercise, and de Groot and Fagerström describes how participants in a fall prevention program were motivated by expectations of improved balance, physical function and health (40). Although such motivations concerned factors that could be seen as highly relevant to themselves, they could still be seen as extrinsic, meaning that the activity of exercise was perhaps not motivating in itself. A key feature of exergames is that the gaming aspect is intrinsically motivating, and that players engage in them longer (41). This was showed by Meldrum and co-authors in a RCT comparing virtual reality exercises with conventional exercises in people with vestibular disorders, where there were no significant differences in functional outcomes between groups, but the VR-exercise group exercised more than the protocol specified and also reported higher enjoyment (42). The participants in our study described the game with words like “pleasant”, “enjoyable”, “fun” and as a “positive experience”. They also pointed out the competitive aspect, where performance is rewarded with scores and points, as motivating (43). Some participants said that to them, exercising should include social interaction, and that playing alone was not motivating. This is in line with the findings of Kaos and co-authors, who found that exergame adherence was higher when playing in a multiplayer setting, than in a single player setting. The authors point out group belonging as crucial to game designs (44). Also, some participants expressed that the games could have been more challenging, both game-wise but also physically. A common sentiment among the participants was that the game was not physically demanding. This is reflected in how the average Borg scale value was around 10, and the average maximal heart rate was marginally higher than 100 BPM, for all three levels (Table 3). Consequently, the game failed to induce any substantial exercise effects. This is in contrast to the systematic review of Peng and co-authors, who found that active video games facilitated light-to-moderate energy expenditure during playing (45). It should be noted that maintaining the player's safety was an important priority for the design of the game in this study, which in turn made it challenging to adequately individualize the difficulty setting for each player. In hindsight, having a software feature which could have dynamically adapted the difficulty level of the movements based on a player's performance during the game would probably be more beneficial than the fixed difficulty setting created in the design process. As such, the game may be better suited for frailer older adults with more pronounced deconditioning and mobility issues, which was also suggested by the participants. Alternatively, the game could also be seen as a way to break up lengthy periods of sitting or sedentary behavior, which can be detrimental to health (46).

With regards to game design, the participants expressed that they would have liked the game to be more challenging, and with more opportunities for progression, which is important in game design; when games become too easy, it is easy to lose motivation (43). The participants suggested using more balloons, at different levels, and perhaps also using props, such as rackets, to reach the objects. The environment in itself was perceived as discomforting by some of the participants, such as a feeling of “being pulled outwards” when standing in an exposed spot. Simeonov and co-authors found that height exposure was perceived as equally stressful in real and virtual environments (47), but Bovim and co-authors found that while gait became unsteady and cautious while walking onto a virtual glass bridge over a high drop, gait normalized relatively quickly, suggesting that people can adapt well to height exposure (48).

One of the aims of the study was to explore whether playing the game would facilitate more physical activity also outside of the game setting, the rationale being that inactive people would find playing the game motivating and thereby being more physically active, and that this would transfer to other physical activities. Most participants expressed that playing the game did not make them more physically active in general and in their daily lives. This can also be seen in the count of daily steps, which was more or less the same before the intervention, during the intervention and after the intervention (Table 6). We have found no studies investigating how playing an exergame may transfer to being more physically active outside of the gaming context, but it can possibly be assumed that feeling fit and as an “exerciser” would be associated with more exercise and physical exertion in daily life, not unlike how Rodrigues and co-authors show in a study of young and middle-aged adults, that past positive exercise experiences are associated with future exercise (49). In the present study, the exercise intervention lasted only two weeks, and was perceived as low intensity, and it is unlikely that it would have any lasting impact on the physical activity behavior of the participants.

This study is limited by a low number of participants. Furthermore, although they were recruited on the basis of being physically inactive, their physical functioning was good, as shown in Table 2. A recurring comment in the interviews was that the game was too easy and that the participants did not feel that they exerted themselves, and the target group for inclusion should likely have been individuals with lower physical capacity and more ADL dependence.

A strength of the study is that we got deeper knowledge about the experiences with the game through the interviews, which will be very important for further game development. The game was developed in close collaboration with older adults, and their input was taken into consideration, from using balloons as the targets to hit, to specifically composed music. This study is a continuation of the process of developing the game optimally for potential target groups.

## 5 Conclusion

In this study we included ten relatively healthy but inactive older adults, where all piloted a VR game with six sessions over two weeks. The main finding of the study is that while older adults enjoyed the experience of playing a virtual reality exergame, the game was not physically demanding enough for the participants, and also that the game presented too few challenges. Game development should focus on tasks that keep players interested and engaged over longer times, without jeopardizing safety.

## Data Availability

The datasets presented in this article are not readily available because participants did not consent to the interview transcripts being available. Requests to access the datasets should be directed to bard.e.bogen@uib.no.

## Footnotes

1
https://www.who.int/news-room/fact-sheets/detail/ageing-and-health

## References

[1] European Union. Demography of Europe—2024 Edition (2024). Available online at: https://ec.europa.eu/eurostat/web/interactive-publications/demography-2024#population-change (Accessed October 28, 2025).

[2] Ding D Nguyen B Nau T Luo M Del Pozo Cruz B Dempsey PC Daily steps and health outcomes in adults: a systematic review and dose-response meta-analysis. Lancet Public Health. (2025) 10(8):e668–e81. 10.1016/S2468-2667(25)00164-1 40713949

[3] Zhao W Hu P Sun W Wu W Zhang J Deng H Effect of physical activity on the risk of frailty: a systematic review and meta-analysis. PLoS One. (2022) 17(12):e0278226. 10.1371/journal.pone.0278226 36454790 PMC9714708

[4] Zhao J Ke Z Huang R Wen X Liu W Wang S Physical activity and the risk of developing 8 age-related diseases: epidemiological and Mendelian randomization studies. Eur Rev Aging Phys Act. (2024) 21(1):24. 10.1186/s11556-024-00359-2 39294593 PMC11412029

[5] Del Pozo Cruz B Ahmadi M Naismith SL Stamatakis E. Association of daily step count and intensity with incident dementia in 78 430 adults living in the UK. JAMA Neurol. (2022) 79(10):1059–63. 10.1001/jamaneurol.2022.2672 36066874 PMC9449869

[6] Kistler-Fischbacher M Weeks BK Beck BR. The effect of exercise intensity on bone in postmenopausal women (part 2): a meta-analysis. Bone. (2021) 143:115697. 10.1016/j.bone.2020.115697 33357834

[7] Sherrington C Fairhall N Kwok W Wallbank G Tiedemann A Michaleff ZA Evidence on physical activity and falls prevention for people aged 65+ years: systematic review to inform the WHO guidelines on physical activity and sedentary behaviour. Int J Behav Nutr Phys Act. (2020) 17(1):144. 10.1186/s12966-020-01041-3 33239019 PMC7689963

[8] Hyde ET Brown DR Webber BJ Piercy KL Omura JD Rose K Meeting the aerobic and muscle-strengthening physical activity guidelines among older US adults, national health interview survey 1998–2018. J Appl Gerontol. (2024) 43(8):1003–14. 10.1177/07334648241232930 38375621 PMC11305966

[9] Crombie IK Irvine L Williams B McGinnis AR Slane PW Alder EM Why older people do not participate in leisure time physical activity: a survey of activity levels, beliefs and deterrents. Age Ageing. (2004) 33(3):287–92. 10.1093/ageing/afh089 15082435

[10] Collado-Mateo D Lavín-Pérez AM Peñacoba C Del Coso J Leyton-Román M Luque-Casado A Key factors associated with adherence to physical exercise in patients with chronic diseases and older adults: an Umbrella review. Int J Environ Res Public Health. (2021) 18(4):2023. 10.3390/ijerph18042023 33669679 PMC7922504

[11] Colder Carras M Van Rooij AJ Spruijt-Metz D Kvedar J Griffiths MD Carabas Y Commercial video games as therapy: a new research agenda to unlock the potential of a global pastime. Front Psychiatry. (2017) 8:300. 10.3389/fpsyt.2017.00300 29403398 PMC5786876

[12] Perez-Marcos D. Virtual reality experiences, embodiment, videogames and their dimensions in neurorehabilitation. J Neuroeng Rehabil. (2018) 15(1):113. 10.1186/s12984-018-0461-0 30477527 PMC6258149

[13] de Bruin ED Schoene D Pichierri G Smith ST. Use of virtual reality technique for the training of motor control in the elderly. Some theoretical considerations. Z Gerontol Geriatr. (2010) 43(4):229–34. 10.1007/s00391-010-0124-7 20814798

[14] Vogt S Skjæret-Maroni N Neuhaus D Baumeister J. Virtual reality interventions for balance prevention and rehabilitation after musculoskeletal lower limb impairments in young up to middle-aged adults: a comprehensive review on used technology, balance outcome measures and observed effects. Int J Med Inform. (2019) 126:46–58. 10.1016/j.ijmedinf.2019.03.009 31029263

[15] Rytterström P Strömberg A Jaarsma T Klompstra L. Exergaming to increase physical activity in older adults: feasibility and practical implications. Curr Heart Fail Rep. (2024) 21(4):439–59. 10.1007/s11897-024-00675-9 39023808 PMC11333506

[16] Herold F Hamacher D Schega L Müller NG. Thinking while moving or moving while thinking—concepts of motor-cognitive training for cognitive performance enhancement. Front Aging Neurosci. (2018) 10:228. 10.3389/fnagi.2018.00228 30127732 PMC6089337

[17] Chen Y Zhang Y Guo Z Bao D Zhou J. Comparison between the effects of exergame intervention and traditional physical training on improving balance and fall prevention in healthy older adults: a systematic review and meta-analysis. J Neuroeng Rehabil. (2021) 18(1):164. 10.1186/s12984-021-00917-0 34819097 PMC8611920

[18] Stanmore E Stubbs B Vancampfort D de Bruin ED Firth J. The effect of active video games on cognitive functioning in clinical and non-clinical populations: a meta-analysis of randomized controlled trials. Neurosci Biobehav Rev. (2017) 78:34–43. 10.1016/j.neubiorev.2017.04.011 28442405

[19] Mirelman A Rochester L Maidan I Del Din S Alcock L Nieuwhof F Addition of a non-immersive virtual reality component to treadmill training to reduce fall risk in older adults (V-TIME): a randomised controlled trial. Lancet. (2016) 388(10050):1170–82. 10.1016/S0140-6736(16)31325-3 27524393

[20] Chen X Wu L Feng H Ning H Wu S Hu M Comparison of exergames versus conventional exercises on the health benefits of older adults: systematic review with meta-analysis of randomized controlled trials. JMIR Serious Games. (2023) 11:e42374. 10.2196/42374 37347534 PMC10337432

[21] LaViola JR J Kruijff E McMahan R Bowman D Poupyrev I. 3D User Interfaces. Theory and Practice. Second Edition ed: Pearson Education (2017).

[22] Ventura S Brivio E Riva G Baños RM. Immersive versus non-immersive experience: exploring the feasibility of memory assessment through 360° technology. Front Psychol. (2019) 10:2509. 10.3389/fpsyg.2019.02509 31798492 PMC6868024

[23] FX C. Virtual Reality Activities for Seniors: Bringing the Beneficial Cognitive, Social and Physical Effects of Immersive Training to the High-functioning Senior Home­-User. Zurich, Switzerland (2023). Available online at: https://www.cosophy.com/ (Accessed October 28, 2025).

[24] Merkel S Kucharski A. Participatory design in gerontechnology: a systematic literature review. Gerontologist. (2019) 59(1):e16–25. 10.1093/geront/gny034 29788319

[25] Song X Ali NM Mhd Salim MH Rezaldi MY. A literature review of virtual reality exergames for older adults: enhancing physical, cognitive, and social health. Appl Sci. (2025) 15(1):351. 10.3390/app15010351

[26] Vanden Abeele V Schraepen B Huygelier H Gillebert C Gerling K Ee R. Immersive virtual reality for older adults: empirically grounded design guidelines. ACM Trans Access Comput. (2021) 14:1–30. 10.1145/3470743

[27] DeSmet A Thompson D Baranowski T Palmeira A Verloigne M De Bourdeaudhuij I. Is participatory design associated with the effectiveness of serious digital games for healthy lifestyle promotion? A meta-analysis. J Med Internet Res. (2016) 18(4):e94. 10.2196/jmir.4444 27129447 PMC4867751

[28] Healy D Flynn A Conlan O McSharry J Walsh J. Older adults’ experiences and perceptions of immersive virtual reality: systematic review and thematic synthesis. JMIR Serious Games. (2022) 10(4):e35802. 10.2196/35802 36472894 PMC9768659

[29] Szczepocka E Mokros Ł Kazmierski J Nowakowska K Łucka A Antoszczyk A The effectiveness of virtual reality-based training on cognitive, social, and physical functioning in high-functioning older adults (CoSoPhy FX): 2-arm, parallel-group randomized controlled trial. JMIR Res Protoc. (2024) 13:e53261. 10.2196/53261 38837194 PMC11187518

[30] SenopiVR. (2023). SenopiMed. Available online at: https://www.senopi.com/products (Accessed October 28, 2025).

[31] Cruz-Jentoft AJ Bahat G Bauer J Boirie Y Bruyère O Cederholm T Sarcopenia: revised European consensus on definition and diagnosis. Age Ageing. (2019) 48(1):16–31. 10.1093/ageing/afy169 30312372 PMC6322506

[32] Podsiadlo D Richardson S. The timed “up & go": a test of basic functional mobility for frail elderly persons. J Am Geriatr Soc. (1991) 39(2):142–8. 10.1111/j.1532-5415.1991.tb01616.x 1991946

[33] Whitney SL Wrisley DM Marchetti GF Gee MA Redfern MS Furman JM. Clinical measurement of sit-to-stand performance in people with balance disorders: validity of data for the five-times-sit-to-stand test. Phys Ther. (2005) 85(10):1034–45. 10.1093/ptj/85.10.1034 16180952

[34] Peters DM Fritz SL Krotish DE. Assessing the reliability and validity of a shorter walk test compared with the 10-meter walk test for measurements of gait speed in healthy, older adults. J Geriatr Phys Ther. (2013) 36(1):24–30. 10.1519/JPT.0b013e318248e20d 22415358

[35] Williams N. The borg rating of perceived exertion (RPE) scale. Occup Med (Chic Ill). (2017) 67(5):404–5. 10.1093/occmed/kqx063

[36] Tudor-Locke C Johnson WD Katzmarzyk PT. Relationship between accelerometer-determined steps/day and other accelerometer outputs in US adults. J Phys Act Health. (2011) 8(3):410–9. 10.1123/jpah.8.3.410 21487141

[37] Garcia Oliveira S Lourenço Nogueira S Alex Matos Ribeiro J Carnaz L Regina Rocha Urruchia V Alcantara CC Concurrent validity and reliability of an activity monitoring for rehabilitation (AMoR) platform for step counting and sitting/lying time in post-stroke individuals. Top Stroke Rehabil. (2022) 29(2):103–13. 10.1080/10749357.2021.1886639 33605190

[38] Hergenroeder AL Barone Gibbs B Kotlarczyk MP Kowalsky RJ Perera S Brach JS. Accuracy of objective physical activity monitors in measuring steps in older adults. Gerontol Geriatr Med. (2018) 4:2333721418781126. 10.1177/2333721418781126 29977979 PMC6024488

[39] Rowland E Conolly A. A worked example of contextualising and using reflexive thematic analysis in nursing research. Nurse Res. (2024) 32(4):17–27. 10.7748/nr.2024.e1924 39206491

[40] de Groot GC Fagerström L. Older adults’ motivating factors and barriers to exercise to prevent falls. Scand J Occup Ther. (2011) 18(2):153–60. 10.3109/11038128.2010.487113 20545467

[41] Buyle M Jung Y Pavlou M Gonzalez SC Bamiou DE. The role of motivation factors in exergame interventions for fall prevention in older adults: a systematic review and meta-analysis. Front Neurol. (2022) 13:903673. 10.3389/fneur.2022.903673 35989930 PMC9388774

[42] Meldrum D Herdman S Vance R Murray D Malone K Duffy D Effectiveness of conventional versus virtual reality-based balance exercises in vestibular rehabilitation for unilateral peripheral vestibular loss: results of a randomized controlled trial. Arch Phys Med Rehabil. (2015) 96(7):1319–28.e1. 10.1016/j.apmr.2015.02.032 25842051

[43] Altamimi R Skinner G. A survey of active video game literature from theory to technological application. Int J Comp Inform Technol. (2012) 1:2277–764.

[44] Kaos MRR Hämäläinen P Graham TCN. Social play in an exergame: how the need to belong predicts adherence. Proceedings of the 2019 Computer Human Interface Conference on Human Factors in Computing Systems (2019).

[45] Peng W Lin JH Crouse J. Is playing exergames really exercising? A meta-analysis of energy expenditure in active video games. Cyberpsychol Behav Soc Netw. (2011) 14(11):681–8. 10.1089/cyber.2010.0578 21668370

[46] Benatti FB Ried-Larsen M. The effects of breaking up prolonged sitting time: a review of experimental studies. Med Sci Sports Exerc. (2015) 47(10):2053–61. 10.1249/MSS.0000000000000654 26378942

[47] Simeonov PI Hsiao H Dotson BW Ammons DE. Height effects in real and virtual environments. Hum Factors. (2005) 47(2):430–8. 10.1518/0018720054679506 16170948

[48] Bovim LP GB Mæland S Aaslund MK Bogen BE. Immediate Gait Adaptation from Walking on a Treadmill to Walking on a Treadmill in a Fully Immersive Virtual Environment. Prague, Czech Republic: European College of Sports Sciences (2019).

[49] Rodrigues F Teixeira DS Neiva HP Cid L Monteiro D. Understanding exercise adherence: the predictability of past experience and motivational determinants. Brain Sci. (2020) 10(2):98. 10.3390/brainsci10020098 32059352 PMC7071831

